# Real world experience of the treatment outcome between photodynamic therapy combined with ranibizumab and aflibercept monotherapy in polypoidal choroidal vasculopathy

**DOI:** 10.1038/s41598-021-99634-w

**Published:** 2021-10-11

**Authors:** I-Hsin Ma, Yun Hsia, Yi-Ting Hsieh, Tzyy-Chang Ho, Tso-Ting Lai, Chung-May Yang, Chang-Hao Yang

**Affiliations:** 1 Department of Ophthalmology, National Taiwan University Hospital, 7 Zhongshan S. Rd., Zhongzheng Dist., Taipei, 10002 Taiwan; 2 Department of Ophthalmology, National Taiwan University Hospital, Hsin-Chu Branch, Hsinchu, Taiwan; 3 Department of Ophthalmology, College of Medicine, National Taiwan University, Taipei, Taiwan

**Keywords:** Biomarkers, Diseases, Medical research

## Abstract

To provide real-world experiences of treating polypoidal choroidal vasculopathy (PCV) patients with photodynamic therapy (PDT) plus intravitreal injection of ranibizumab or intravitreal injection of aflibercept alone. Retrospective chart review of patients with PCV in a single tertiary referral center in Taiwan. Chart review of PCV patients treated with PDT and injection of ranibizumab or injection of aflibercept. A total of 101 eyes of 101 patients (38 females and 63 males) were reviewed. Of those, 48 and 53 eyes received primary/adjunctive PDT along with injections of ranibizumab or intravitreal injections of aflibercept only, respectively. Initial visual acuity (VA) and central subfield choroidal thickness were similar between the two groups (*p* > 0.05). In addition, changes in VA at 3, 6, and 12 months post treatment were similar. The central retinal thickness decreased with either treatment (*p* < 0.01); however, this change did not translate into VA performance (*p* > 0.05). In the subgroup analysis of pachychoroid and non-pachychoroid patients, better initial VA and post-treatment VA at 3 months and 6 months was noted in the latter group of patients treated with anti-vascular endothelial growth factor monotherapy (*p* < 0.05). Aflibercept monotherapy is comparable with PDT plus ranibizumab in PCV patients with PCV (pachychoroid and non-pachychoroid patients). In addition, better prognosis regarding VA was observed in non-pachychoroid patients treated with aflibercept monotherapy.

## Introduction

Polypoidal choroidal vasculopathy (PCV) is a subtype of age-related macular disease, characterized by dilated choroidal vasculature with bulbs and leaking sites, and the neovascular complex was usually sub-retinal pigment epithelium, commonly associated with subretinal exudation and hemorrhage^1–4^. In the Asian population, the prevalence of PCV was markedly higher than that of age-related macular degeneration, with male preponderance, and clinical characteristics such as macular location of abnormal vessels and more unilateral involvement^2,3,5,6^.

Considering the high prevalence and possible drastic visual debilitation, research on the treatment of PCV is warranted. Photodynamic therapy (PDT) has been the mainstay of PCV therapy for many years. Nevertheless, numerous studies advocate therapy with injections of anti-vascular endothelial growth factor (anti-VEGF)^7–12^. Treatment with anti-VEGF therapy alone is effective in reducing the fluid from PCV lesions and improving visual acuity (VA); however, it is less efficient against polypoidal lesions^13–15^. Combination therapy, with two or three combined protocols, has also been suggested by various research groups^8,14,16–21^. The EVEREST II study revealed a higher rate of polyp regression at 6 months post treatment with PDT combined with injection of ranibizumab^22^. The PLANET study disclosed similar outcomes in terms of polyp size and activity, central retinal subfield thickness, and VA at 1 year post treatment^23^. Based on the availability of multiple treatment options, physicians may tailor the disease management to individual patients with different clinical characteristics. In addition, the efficacy of PDT in combination with ranibizumab and that of aflibercept monotherapy have not been evaluated. Consequently, the criteria for the selection of the most appropriate treatment are under debate.

A treatment protocol was suggested by a Japanese and Taiwanese expert summit based on presenting VA and treatment response^24^. Despite using VA as a subjective indicator of disease activity, the other disease manifesting foci may be the choroidal thickness and fluid accumulation presented and measured using optical coherence tomography (OCT) in patients with PCV. The effects of choroidal characteristics, such as choroidal hyperpermeability, subfoveal choroidal thickness and the treatment effects with anti-VEGF or with PDT have been discussed extensively^21,25,26^. Based on these data, we investigated the prognosis of VA in patients receiving various treatments using different OCT biomarkers.

## Methods

### Enrollment criteria

Treatment-naïve patients diagnosed with PCV at the National Taiwan University Hospital (NTUH) from 2015 to 2018 were enrolled for review. Diagnostic criteria were the detection of polyps using indocyanine green angiography (ICGA) and early dye pooling. Two retina specialists independently reviewed the examination films and confirmed the diagnosis. Patients with proliferative diabetic retinopathy, myopic maculopathy, previous rhegmatogenous retinal detachment, or other causes of choroidal neovascularization were excluded. The study was approved by the institutional review board of the National Taiwan University Hospital, informed consent was obtained from all subjects and all investigations were conducted in accordance with the tenets of the Declaration of Helsinki.

### Treatment protocols

Patients received different therapies according to the preferences of the physician and patient at the time of treatment initiation. In the PDT plus ranibizumab group, patients received initial intravitreal ranibizumab (IVR) injection at 0.5 mg per eye, closely followed by standard PDT (verteporfin: 6 mg/m^2^; full laser irradiance: 600 mW/cm^2^; treatment time: 83 s) within 1 week; the second injection of ranibizumab was administered 1 month after the initial IVR. PDT was applied to all the branching vascular network and polyp areas demonstrated by ICGA. Additional anti-VEGF injections were continued at a *pro re nata* basis (with intervals of ≥ 28 days for each injection) according to persistent disease activity [i.e., subretinal hemorrhage, sub-retinal pigment epithelium hemorrhage, intraretinal cysts, subretinal fluid (SRF) or serous retinal-pigment epithelial detachment (sPED)] noted through follow-up OCT. Rescue therapy with PDT was performed only in patients with persistent or newly developed polypoidal lesions plus disease activity, with an interval of ≥ 3 months from the previous PDT.

In the intravitreal aflibercept (IVA) injection alone group, the protocol included three monthly injections followed by *pro re nata* injection according to disease activity revealed using OCT, with an interval of ≥ 28 days.

### Measurement parameters

Demographic data, best-corrected visual acuity (BCVA) as logarithm of the minimum angle of resolution (logMAR), and subfield choroidal thickness at initial diagnosis were recorded. The BCVA was recorded at 3, 6, and 12 months post the initial treatment (either anti-VEGF or PDT). The total number of injections at 12 months was also documented. Pachychoroid was defined as: (1) enlarged choroidal vessels detected using OCT with compression of choriocapillaris; (2) subfoveal choroidal thickness > 267.5 µm; and (3) dilated, hyperpermeable choroidal vessels detected using ICGA^27–30^. In this study, patients who met two of these three criteria were classified into the pachychoroid subtype. Other documented OCT biomarkers were SRF, intraretinal fluid (IRF) or cysts, and serous PED (sPED).

### Statistical analysis

Primary outcomes of this study were post-treatment BCVA and CRT between groups. Secondary outcomes were post-treatment BCVA and CRT in two subgroups categorized by choroidal characteristics.

Differences between demographic data, BCVA, changes in VA, central retinal thickness (CRT), changes in CRT, and the numbers of intravitreal injections were analyzed using *t* test. Sex and OCT biomarkers (SRF, IRF, and sPED) were analyzed using chi-squared test. For the evaluation of choroidal thickness and selection of treatment modality, a subgroup analysis by *t* test was performed with stratification of subfoveal choroidal thickness to compare the two treatment protocols. The statistical analysis was performed in R (V.4.0.3), and *p* < 0.05 denoted statistical significance.

## Results

### Demographics between PDT plus ranibizumab and aflibercept alone

A total of 101 eyes of 101 patients (38 females and 63 males) were included for review. Of those, 48 and 53 received primary/adjunctive PDT along with IVR injection and IVA injections alone, respectively. The average age of patients in these groups was 68.83 ± 7.42 years and 64.43 ± 9.55 years, respectively (*p* = 0.01). The initial VA was logMAR 0.63 ± 0.42 and 0.57 ± 0.40 (*p* = 0.45); central subfield choroidal thickness was 226.41 ± 96.14 µm and 259.98 ± 93.01 µm (*p* = 0.10); and CRT was 301.26 ± 96.31 µm and 306.72 ± 95.08 µm (*p* = 0.79), respectively. Regarding the OCT biomarkers, the presence of fluid accumulation at the subretinal location was similar between the two groups; however, the percentage of IRF was lower in the IVA group, while that of sPED was lower in the PDT plus IVR group (Table 1).

**Table 1:** Demographics of patients with PCV receiving different treatments.

	Group 1 (n = 48)	Group 2 (n = 53)	*p* value
Age, years	68.83 ± 7.42	64.43 ± 9.55	0.01*
**Sex**			0.29
Male	33	30	
Female	15	23	
Baseline BCVA	0.63 ± 0.42	0.57 ± 0.40	0.45
Baseline SFCT	226.41 ± 96.14	259.98 ± 93.01	0.10
Baseline CRT	301.26 ± 96.31	306.72 ± 95.08	0.79
**Baseline OCT biomarkers**			
SRF	37 (77.1%)	36 (67.9%)	0.42
IRF	16 (33.3%)	6 (11.3%)	0.01*
sPED	24 (50.0%)	38 (71.7%)	0.04*
Total number of IVI	3.57 ± 2.14	3.32 ± 1.88	0.55
Total number of PDT	1.53 ± 0.83		

Values are presented as the mean ± standard deviation or number (percentage).

*Group 1* intravitreal injection of ranibizumab + photodynamic therapy, *Group 2* intravitreal injection of aflibercept, *BCVA* best-corrected visual acuity, *CRT* central retinal thickness, *IRF* intraretinal fluid, *IVI* intravitreal injection, *OCT* optical coherence tomography, *PCV* polypoidal choroidal vasculopathy, *PDT* photodynamic therapy, *SFCT* subfoveal choroidal thickness, *sPED* serous pigment epithelial detachment, *SRF* subretinal fluid.

*Indicates chi-squared test; otherwise, *t* test.

### Treatment and VA

**Figure 1:**
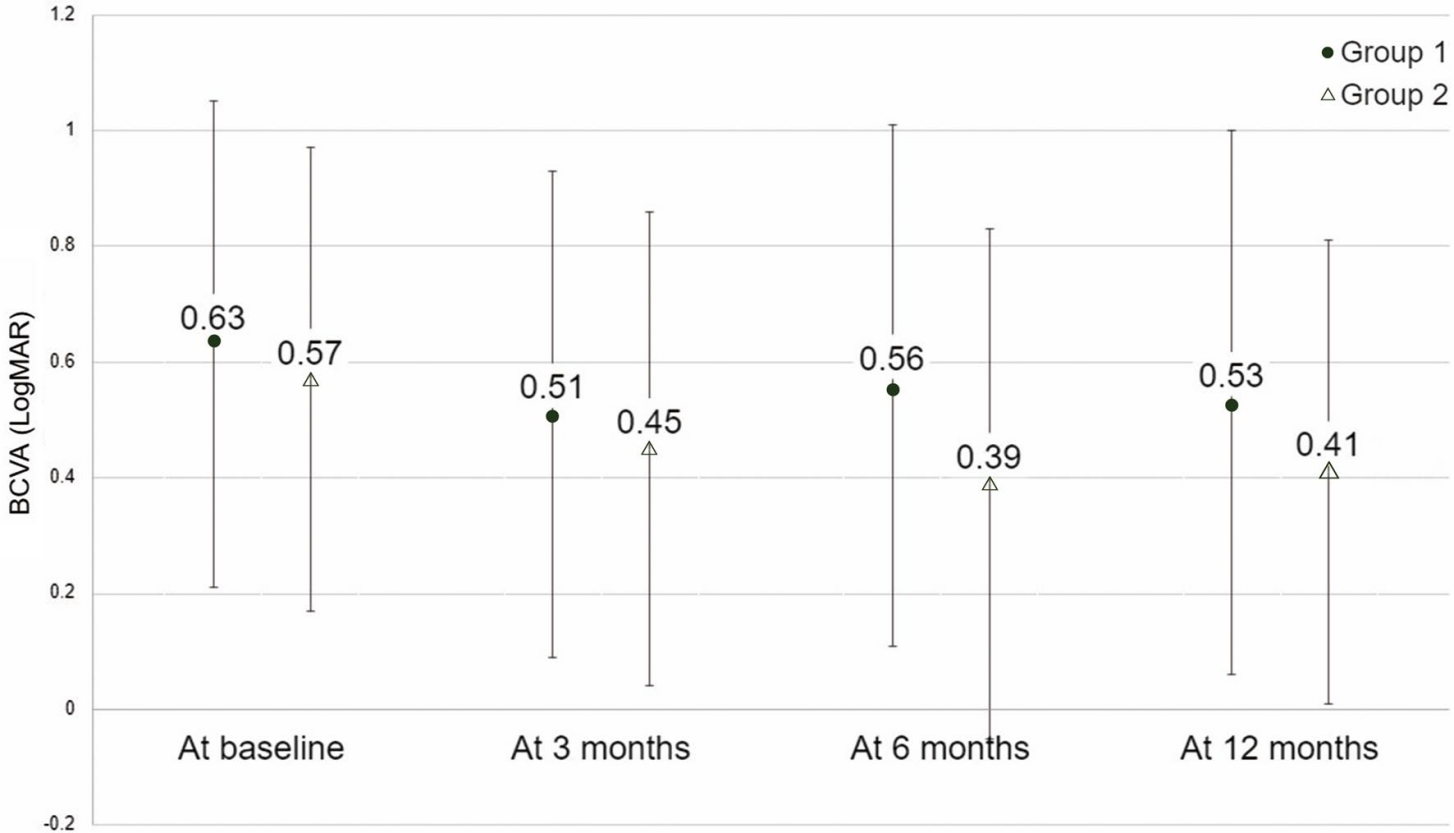
Mean visual acuity in the two treatment groups at different timepoints. *Group 1* intravitreal injection of ranibizumab + photodynamic therapy, *Group 2* intravitreal injection of aflibercept alone, *BCVA* best-corrected visual acuity, *LogMAR* logarithm of the minimum angle of resolution.

**Figure 2:**
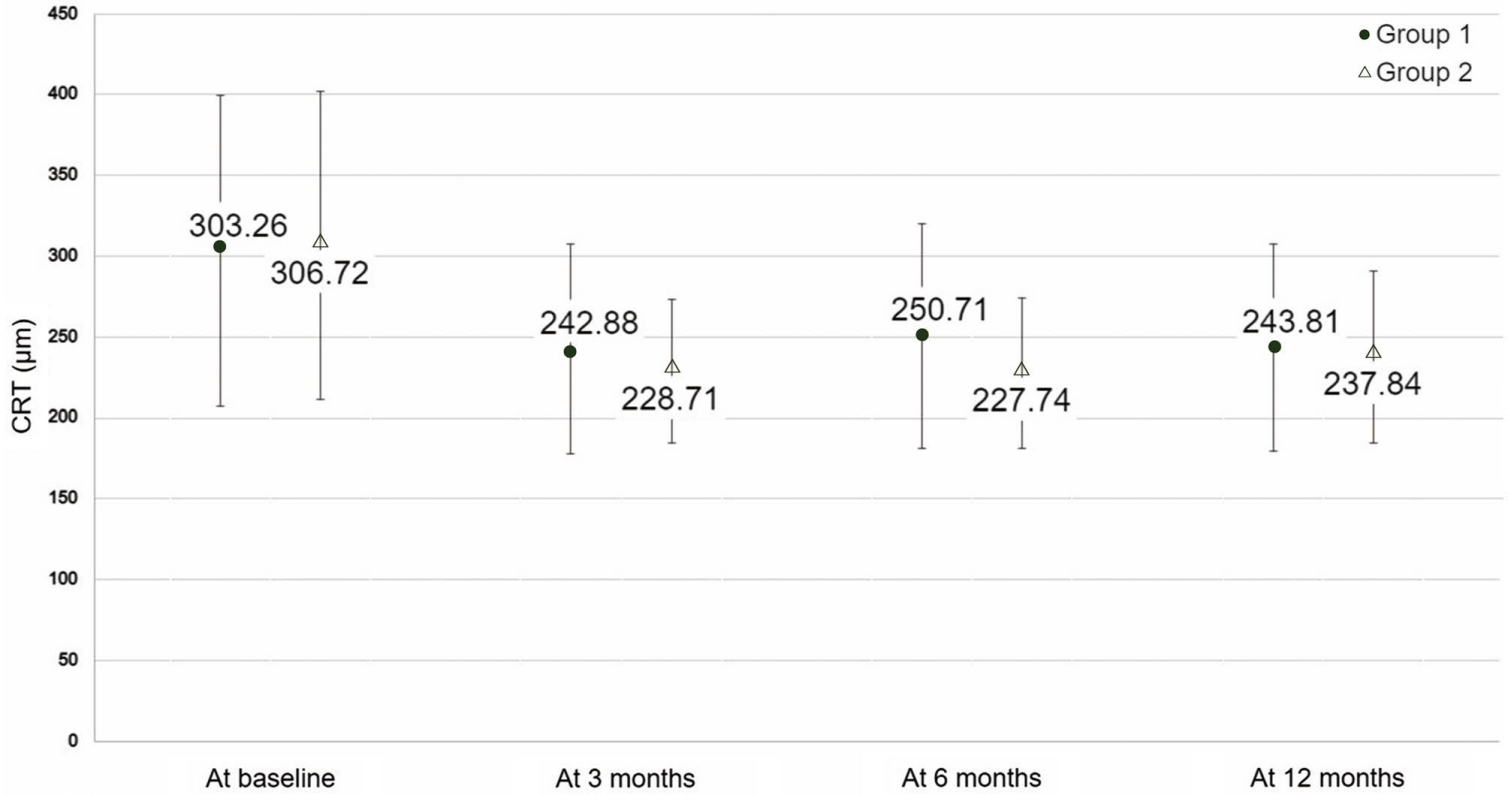
Mean central retinal thickness in the two treatment groups at different timepoints. *Group 1* intravitreal injection of ranibizumab + photodynamic therapy, *Group 2* intravitreal injection of aflibercept alone, *CRT* central retinal thickness.

Although not statistically significant, the mean VA gradually improved with time regardless of the treatment modality. In addition, the changes in VA observed at 3, 6, and 12 months post treatment did not differ between the groups (Figs. 1, 2). At the initial assessment, OCT biomarkers exhibited a bias towards a lower percentage of IRF in the IVA group. Therefore a subgroup analysis in patients with IRF at baseline was carried out, which did not demonstrate differences in visual outcome between the treatments.

### Treatment and CRT

The mean CRT values at all time points were not statistically different between two groups (*p* > 0.05); however, in both treatment groups, the CRT was significantly decreased at 3, 6, and 12 months post treatment (*p* < 0.05). (Fig. 2). The decrease in CRT observed at 6 month was more significant in the combination group, but was not sustained until 12 months (Table 2). The presence of IRF didn’t affect the findings stated above. Both treatments exerted similar effects on this subpopulation.

**Table 2:** Post-treatment effect in different groups.

	Group 1	Group 2	*p* value
Baseline BCVA	0.64 ± 0.42	0.57 ± 0.40	0.45
BCVA at 3 months	0.51 ± 0.42	0.45 ± 0.41	0.51
BCVA at 6 months	0.56 ± 0.45	0.39 ± 0.44	0.06
BCVA at 12 months	0.54 ± 0.47	0.41 ± 0.40	0.15
Baseline CRT	301.26 ± 96.31	306.72 ± 95.08	0.79
CRT at 3 months	242.88 ± 65.00	228.71 ± 44.47	0.25
CRT at 6 months	250.71 ± 69.36	227.74 ± 46.41	0.08
CRT at 12 months	243.81 ± 63.94	237.84 ± 53.21	0.65

Values are presented as the mean ± standard deviation.

*Group 1* intravitreal injection of ranibizumab + photodynamic therapy, *Group 2* intravitreal injection of aflibercept alone, *BCVA* best-corrected visual acuity, *CRT* central retinal thickness.

### Choroidal characteristics, treatment option, and outcome

After stratifying the patients into pachychoroid and non-pachychoroid sub-populations, statistical analyses were carried out to determine the anatomical and functional outcomes of both treatment options. Regarding demographics, non-pachychoroid patients were significantly older than pachychoroid patients (*p* < 0.05).

In terms of treatment prognosis, regardless of the therapeutic options, patients with pachychoroid had better outcome in VA at all follow-up time points, although the differences did not reach statistical significance. Stratified the patients into pachy- and non-pachychoroid subgroups, the numbers of patient received combination therapy was 12, and monotherapy was 25 in the pachychoroid group; whereas the numbers were 33 and 28, respectively, in the non-pachychoroid group. In pachychoroid patients, aflibercept monotherapy exerted a slightly greater retinal drying effect, with marked decrease in CRT compared with the PDT plus IVR injection; however, the difference did not reach statistical significance. Moreover, in these patients, there was no difference between the treatments in visual function. In non-pachychoroid patients, the decrease in CRT did not differ significantly between treatments. However, the VA was better throughout the treatment period in patients who received aflibercept monotherapy, and reached statistical significance at 3 and 6 months post treatment (*p* < 0.05) (Table 3). To note, the initial VA was better in the monotherapy group of the non-pachychoroid patients as well.

**Table 3:** Therapeutic effects in PCV with varied choroidal thickness.

	Pachychoroidal (n = 37)			Non-pachychoroidal (n = 61)			*p* value (overall)
	Group 1 (n = 12)	Group 2 (n = 25)	*p *value	Group 1 (n = 33)	Group 2 (n = 28)	*p* value	
Age	66.82 ± 8.23	61.43 ± 8.82	0.02	70.41 ± 6.49	70.28 ± 8.29	0.95	.0001
BCVA baseline	0.56 ± 0.41	0.64 ± 0.45	0.48	0.70 ± 0.43	0.44 ± 0.26	0.02	0.83
SFCT baseline	240.71 ± 111.47	279.39 ± 89.71	0.19	209.19 ± 78.64	222.23 ± 89.91	0.65	0.01
BCVA 3 months	0.45 ± 0.45	0.51 ± 0.47	0.63	0.56 ± 0.4	0.33 ± 0.23	0.03	0.66
BCVA 6 months	0.48 ± 0.45	0.41 ± 0.49	0.62	0.63 ± 0.45	0.32 ± 0.28	0.01	0.46
BCVA 12 months	0.47 ± 0.51	0.4 ± 0.43	0.65	0.59 ± 0.44	0.41 ± 0.34	0.15	0.34
CRT baseline	294.43 ± 94.95	302.3 ± 99.02	0.78	300.32 ± 95.64	315 ± 89.74	0.62	0.72
CRT 3 months	256.77 ± 69.93	223.38 ± 49.58	0.08	226.29 ± 58.31	239.9 ± 30.4	0.38	0.44
CRT 6 months	253.73 ± 80.65	217.76 ± 37.4	0.07	241.21 ± 54.61	248.7 ± 57.88	0.73	0.6
CRT 12 months	236 ± 59.84	223 ± 47.42	0.43	244.38 ± 63.96	266.18 ± 54.17	0.3	0.11
Number of IVI (1 year)	3.05 ± 1.83	3.57 ± 2.03	0.33	4 ± 2.36	2.83 ± 1.47	0.07	0.86

Values are presented as the mean ± standard deviation.

*Group 1* intravitreal injection of ranibizumab + photodynamic therapy, *Group 2* intravitreal injection of aflibercept alone, *BCVA* best-corrected visual acuity, *CRT* central retinal thickness, *IVI* intravitreal injection, *PCV* polypoidal choroidal vasculopathy.

Recurrence was noticed in 55 patients, 44 received rescue IVI therapy and 3 received PDT. Re-treatment was arranged within 1 month of censored disease reactivation. 8 patients decided not to receive additional treatment.

## Discussion

This study included 101 naïve eyes with PCV treated with PDT plus IVR injection or IVA injection alone. The patients were followed up for ≥ 1 year and received treatment as indicated. The present study found that these treatment options did not differ significantly in terms of VA prognosis and CRT. In addition, the treatment outcome did not differ in pachychoroid patients. Nevertheless, in non-pachychoroid patients, significantly better VA was observed at 3 and 6 months post IVA injection alone versus PDT plus IVR injection. However, the initial VA of the non-pachychoroid patients in the IVA injection alone group was better than that recorded in the PDT plus IVR injection group.

The selection of the optimal treatment for patients with PCV has been under debate. For instance, regression of the branching vascular network and collapse of polyp has been the criterion for the evaluation of the therapeutic effect; however, VA remains the main concern for most patients. PDT has demonstrated better polyp closure effects and longer disease-free intervals^31,32^. Nonetheless, the vessel closure effect raises concerns about possible choroidal infarction after PDT, leading to irreversible retinal atrophy and VA loss; although this approach cures the primary lesion, it has been associated with loss of visual function in certain cases^33,34^. Therefore, non-pachychoroid patients may be more vulnerable. The effects of combination therapy are well documented, and numerous different combination regimens have been proposed^8,14,16–21,35^. In the EVEREST II study, the PDT combination group showed greater improvement in VA and regression of polypoidal lesions than the IVR monotherapy group. Moreover, the total number of IVR injections was lower in the combination group. Safety wise, no severe or sudden vision loss was reported among participants in combination therapy group after PDT treatment. In terms of disease activity, both groups reported less activity and lower hemorrhage rate under regular follow-up and treatment. For anti-VEGF monotherapy, the PLANET study showed good visual and functional response in > 85% of treated patients, with few number of patients requiring adjunct PDT. In a real-world study, anti-VEGF monotherapy and combination therapy had comparable long-term visual outcomes after a follow-up of 6 years^36^. Another study reported comparable visual outcome, but fewer intravitreal injections and longer time of recurrence in the combination group^31^. The present study yielded similar results, with both treatment achieving decrease in CRT and improvement in vision; the differences between the treatments were negligible. In our cohort, there was a low number of intravitreal injections compared with that reported in clinical trials and some studies. This was attributed to the balance between out-of-pocket injections and the limited injections provided under the National Health Insurance coverage. Most retina specialists and patients decided to arrange additional follow-up appointments with *pro re nata* injections instead of monthly or bi-monthly injections.

PCV belonged to the spectrum of pachychoroid diseases. However, pachychoroid is defined by the choroidal thickness and the choroidal vessel configuration noted using OCT, as well as the choroidal vascular permeability presented using ICGA^29^. It has been proposed that the pachychoroid-driven process involves choroidal congestion and hyperpermeability. Choroidal congestion can be easily visualized through structural OCT by the enlarged Hattler and/or Sattler vessels occupying the choriocapillaris layer; notably, the hyperpermeability is best visualized with late-phase ICGA^29,37^. The relationship between choroidal thickness, dilated vessels, and hyperpermeable vasculatures is intriguing; patients with 94% thick choroid PCV showed dilatation of choroidal vessels on OCT; however, only 11–68% of those with thick choroid presented with hyperpermeability observed using ICGA^28^. According to these subgroup disease patterns, researchers hypothesized that PDT may exert a greater effect in patients with pachychoroid. A prospective trial demonstrated that patients with choroidal hyperpermeability required substantially fewer intravitreal injections than those without hyperpermeability when receiving the combination of PDT and anti-VEGF therapy^38^. Choroidal thickness affects treatment outcome was also observed in another retrospective study. In the study, patients treated with PDT were followed for 5 years, and showed better maintenance of VA in the pachychoroid cohort versus the non-pachychoroid cohort^39^. Knowing these differences, we defined our pachychoroid cohort as those who met two of the three aforementioned criteria (including SFCT, choroidal hyperpermeability and enlarged choroidal vasculature visible on OCT), and to further validate the outcome between treatments. As for non-pachychoroid patients, anti-VEGF therapy alone yielded better CRT drying effect as compared with PDT adjunct therapy, however the prognosis for VA varied^30,40^.

The present study yielded similar results to previous studies in non-pachychoroid patients. Patients who received anti-VEGF monotherapy were linked to better prognosis for VA than those who received PDT combination therapy. In addition, the number of injections was lower in the monotherapy group in a *pro re nata*-based clinical setting. In previous studies, PCV patients with thinner SFCT responded better to anti-VEGF monotherapy alone. This was represented by a greater retinal drying effect and thinner CRT. Along with the higher mean age in this group, these patients were considered to be closer to the neovascular age-related maculopathy spectrum^26,30,41^. However, in our cohort, the CRT was not significantly different and only differences in VA were demonstrated. More specifically, the initial VA was also better in this group; thus, this improvement should not be viewed as a pure benefit of the therapy^42,43^. Instead, the need for fewer injections in the anti-VEGF monotherapy group could be attributed to their good response. In the pachychoroid group, limited difference was observed between treatments in terms of VA and CRT. In the monotherapy group, only the number of intravitreal injections was slightly higher and the CRT showed a better decreasing trend, although these differences did not reach statistical significance. This could also be interpreted as the additional effect of the extra intravitreal injections, though the total number of injections was markedly lower than that reported in clinical trials. Overall, the present study demonstrated that PDT plus IVR injection and IVA injection were associated with similar visual prognostic benefits in both subgroups of patients with PCV.

The initial VA in our cohort was worse than that recorded in the EVEREST II and PLANET studies. In the EVEREST II study, the baseline VA was 61.1 letters in Early Treatment Diabetic Retinopathy Study (ETDRS), which corresponds to approximately logMAR 0.48. In the PLANET study, the baseline VA in the IVI monotherapy group was 57.7 letters (ETDRS) and approximately 0.54 when transformed to the logMAR scale^22,23^. In our study, the baseline VA for the PDT plus IVR injection and IVA injection monotherapy groups was 0.63 logMAR and 0.57 logMAR, respectively. The improvements recorded at the end of this study were slightly lower than those reported in previous trials. This difference may be partly attributed to the baseline VA; the final VA was closely correlated with the baseline VA^42,43^. Another key difference was that the number of intravitreal injections in this study was markedly lower than those previously demonstrated in the two aforementioned trials, with average of 3.57 ± 2.14 injections in the combination group and 3.32 ± 1.88 in the monotherapy group. In our study, after the initial treatment (i.e., one PDT session plus two monthly IVR injections and three monthly IVA injections, respectively), further administration of drug was performed on a *pro re nata* basis. Given the National Health Insurance reimbursement plan and the compliance of patients, the overall number of injections was low, and this effect has been observed in almost all real-world treatment studies in Taiwan^21,44^. This could have affected the final VA of the cohort to a certain extent.

The present study revealed that PDT plus IVR injection and IVA injection monotherapy yielded similar functional and anatomical benefits at 1-year follow-up. Although there was limited difference in prognosis noted between the different choroid patterns, the prognosis was not affected by the treatment option. Of note, the selection of treatment modality was based on a mutual decision taken by the retinal specialist and patient rather than based on specific disease biomarkers; this approach provided a neutral basis for analysis. Limitations of this study include its retrospective nature, relatively short follow-up period and lower numbers of injection as compared with large prospective trials. In addition, due to the clinical settings, it was not possible to follow up all patients using ICGA. This limited the study of polyp closure rates. Nevertheless, our study included a large number of patients in both arms, shedding light on future treatment decisions. We propose that the improvement in CRT and maintenance of VA in both treatment groups implies the importance of regular follow-up and treatment when indicated. Although OCT biomarkers and pachychoroid features may be prognostic factors, they may not be sufficient to serve as a marker for the selection of treatment. Lastly, both treatment regimens performed well in our pachychoroid cohort, while IVA injection monotherapy may yield better VA in non-pachychoroid patients.
